# Topics of the Month

**Published:** 1894-02

**Authors:** 


### TOPICS OF THE MONTH.

THE Hospital College of Medicine, of Louisville, which is the Medical Department of Central University of Kentucky, has moved into a splendid new building, located at Preston and Chestnut streets. The dedicatory exercises were held on Tuesday evening, January 9, 1894, at which a large audience was assembled, including nearly 200 matriculates of the college. Prof. John A. Larrabee, M. D., President of the college, conducted the exercises, and around him were seated the members of the faculty, namely: Drs. Dudley S. Reynolds, Frank C. Wilson, Samuel G. Dabney, Thomas Hunt Stucky, James Lewis Howe, John Edwin Hays, H. Horace Grant, Lewis S. McMurtry, and P. Richard Taylor. Besides these were the Rev. T. M. Hawes, and Drs. William Bailey, J. M. Matthews, Sam Cochran, Ap. Morgan Vance, A. Wilkes Smith, of Richmond; H. E. Pelle and B. Allen. Dr. Paul Y. Tupper, Professor of Surgery at the St. Louis Medical College, the guest and orator of the evening, and a former graduate of the Hospital College, was also present.

Dr. Larrabee's address was an interesting and eloquent setting forth of the progress made by the college, and especially did it discourse upon the advantages of higher medical education. We extract from this interesting address the following:

Today clinical teaching has surpassed all other modes of instruction, and the young practitioner is sent to the bed-side with an experience in the management of disease which heretofore required years of practice to obtain.

In adopting the three years' graded course of instruction, the student escapes the intolerable bore of listening to the same set of lectures year after year. I can never forget when, as a beginner in medicine, the nomenclature of disease was rolled in upon me from the chair of practice, and that nearly the whole year was passed in the attempt to follow, intelligently, lectures, the value of which was wholly unappreciated. The gastro-epiploica-dextra and the gastro-epiploica-sinister were mixed with duodinal dyspepsia, and the whole treated by a subnitrate of bismuth.

To the medical students here assembled I mean to say a few words. What a change has passed over the spirit of our dreams! The medical student of today is no longer the Dervish plowing through the town with his slouchy gait like a Bedouin of the desert, but he is a gentleman possessed of refinement, whose ambition is to become a learned scientist. Permit me to give you a little advice in regard to study. Many students fail because they try to grasp too much.

The Louisville Hospital College of Medicine is the first in the South to advance the standard of medical education, and we congratulate the faculty, as well as the friends of this excellent institution, upon such a substantial marking of her progressive career as this splendid new edifice indicates.

---

In our issue for January we commented upon the illustrated calendar, for 1894, issued by the Maltine Manufacturing Company. At that time we were ignorant of the fact that the New York *Medical Journal*, in its issue of December 23, 1893, under the head of "Quousque Tandem?" had criticised the Maltine Company on this subject. The company now asks the publication of its letter to the New York *Medical Journal* in these columns, a privilege that we gladly grant. The letter is as follows:

*To the Editor of the New York Medical Journal:*

Sir—Your reference to our calendar for 1894 demands our attention. While you did not mention us by name, the reference is so direct that the physicians who received the calendar can not but know to whom you referred.

It has been our custom for several years to send to the medical profession, throughout the United States, portraits of eminent physicians and surgeons and, inasmuch as their distribution has been scrupulously confined to medical men of good repute, no objection has been offered by those gentlemen whose likenesses we reproduced. Not a copy of this calendar, nor of any of our other numerous publications, has ever been sent to the laity.

Maltine is distinctly not a "patent medicine," nor has it ever been advertised to the public, and, therefore, we have considered it within our province to distribute portraits just as we have promulgated testimonials from the most eminent physicians and chemists in this country and Europe.

We have statistics to prove that ninety per cent. of the physicians of the United States prescribe maltine. This fact, in addition to the fact that we reach the patient *only through the physician*, would seem to amply vindicate our use of the likeness of a physician whose pictures are on public sale and have continually appeared in the public press, and who is well known as a public man.

The portraits referred to were not used to push the sale of our preparations, as was the portrait of Dr. D. Hayes Agnew, recently published by us. It will be remembered that we printed under Dr. Agnew's portrait a facsimile of his indorsement of maltine. Our only reason for publishing the portrait of Dr. —— was because we thought it would interest his medical brethren, who have shown so high an appreciation of the series of likenesses we have already published.

We should like further to say, that as soon as objection was made by him we suspended the distribution of the calendars, as we would not knowingly offend even one of the honorable profession, to whom we are so greatly indebted.

THE MALTINE MANUFACTURING COMPANY.

NEW YORK, December 26, 1893.

---

THE Jennie Casseday Infirmary for Women, at Louisville, Ky., was enlarged during the past Summer by the addition of a wing, in which there are eight additional rooms for patients and an operating room, thus greatly increasing and improving its facilities. Dr. L. S. McMurtry, the surgeon in-charge, has lately been doing a long series of abdominal operations in this hospital, where he has accepted every case that presented, including in the group many of the most unpromising sort. Among the number may be mentioned perforative appendicitis, multiple abscesses, pyosalpinx, pelvis-bound myomata treated by hysterectomy, and other similar desperate conditions. Only one death has occurred in this group ; that was a case of hysterectomy for myofibroma which had undergone malignant degeneration, and the patient had become a morphine habitué.

---

IT IS announced that physicians in New York City attached to any hospital, under a recent ruling of the courts, can give their testimony in any case of a patient who is an inmate of the institution in which they serve, before a referee, thus avoiding annoyance and loss of time in attending court. This is a wise step towards reforming a great evil, but we think it would be well to extend the privileges by statutory enactment to cover the court attendance of all physicians. It is very annoying for medical men in active practice to dance attendance upon "the law's delay," to the detriment of their patients and to the annihilation of good temper. Lawyers are too indifferent as to the value of physicians' time, and courts are sometimes very unaccommodating in relation to their emergency necessities. A statute compelling counsel to stipulate to take a physician's testimony before a Commissioner would solve a very difficult problem.

Another point of importance that now comes to our mind in this connection, relates to the fees of physicians as experts. Not seldom counsel manage to extract an opinion from a physician who is placed on the witness stand as to fact, thus escaping the payment of compensation, a practice that is reprehensible, if not illegal. Dr. H. N. Moyer, of Chicago, the distinguished neurologist, has done the profession great service in Illinois by demonstrating, as he did in the Prendergast trial, that an expert cannot be compelled to testify in that State without compensation.

---

Dr. Seneca D. Powell, in his inaugural address as President of the Medical Society of the County of New York, made some admirable suggestions, that it is hoped will be adopted, looking to the correction of some existing evils. The first has relation to the practice of many dispensary and hospital associations with reference to the free treatment of patients who are able to pay, at least, a moderate sum for professional services. The service of rich corporations for nominal fees was also condemned. He further suggested the appointment of a commission of competent physicians to pronounce upon the expert testimony given before the courts. The enactment of a statutory law to correct the medical expert evil, and certain other legislation relating to the society, was advocated. These, together with matters of local interest, were ably dealt with by the new president, showing his familiarity with the medical affairs of the society and of the country, and demonstrating the wisdom of his election.

